# Bacterial community profiling in artificial lagoons and groundwater of Qatar using a MALDI-TOF MS approach

**DOI:** 10.1007/s11356-026-37851-4

**Published:** 2026-05-29

**Authors:** Hayat Aljabiry, Elizabeth H. Bailey, Scott D. Young, Sathyavathi Sundararaju, Nabil Zouari

**Affiliations:** 1 Environmental Sciences Program, Department of Biological and Environmental Sciences, College of Arts and Sciences, Qatar University, P.O. Box: 2713, Doha, Qatar; 2 School of Biosciences, University of Nottingham, Sutton Bonington Campus, LE12 5RDN Loughborough, UK; 3 Biomedical Research Centre, Qatar University, P.O. Box: 2713, Doha, Qatar

**Keywords:** Artificial lagoons, Groundwater, Bacterial community, MALDI-TOF MS, Treated wastewater, Qatar

## Abstract

In Qatar, potable water depends largely on desalination, while groundwater and treated wastewater are used for irrigation, with the latter having limited reuse opportunities that have led to the establishment of artificial storage lagoons across the country. Those artificial lagoons can act as microbial reservoirs, raising interest in their potential interaction and possible infiltration into the groundwater aquifers in Qatar. Hence, this study investigated bacterial community dynamics and their possible infiltration in nine lagoons and 13 nearby wells using culture-based methods and MALDI-TOF MS. A total of 150 strains were identified, representing 33 species, dominated by *Pseudomonas* (37%), *Acinetobacter* (15%), *Aeromonas* (12%), and *Enterobacter* (10%), with the remaining 26% from other genera. Both lagoons and wells contained coliform bacteria at levels exceeding regulatory thresholds for irrigation water. Alpha diversity indices showed significantly higher richness and diversity in lagoons compared with wells (Shannon, *p* = 0.038; Simpson, *p* = 0.045; and Chao1, *p* = 0.012), indicating the lagoon open system supports microbial diversity. PCA and GLM analyses highlighted associations between treated wastewater-related bacterial communities in lagoons and adjacent wells, with *P. aeruginosa* found to be abundant across both environments. Protein profile clustering of *E. coli* and *P. aeruginosa* further supported similarities between lagoon and groundwater strains. These findings demonstrate that MALDI-TOF MS can provide rapid and reliable insight into bacterial communities in arid region water systems and highlight the value of expanding microbial monitoring beyond traditional coliform indicators.

**Supplementary Information:**

The online version contains supplementary material available at 10.1007/s11356-026-37851-4.

## Introduction

Investigating the urban water cycles in arid environments is crucial in the strategy of water management, given the lack of freshwater bodies, such as rivers and reservoirs, as well as low rainfall in most arid and semiarid regions. However, water supplies may be significantly provided by three types of water: desalinated water, groundwater, and recycled water (Darwish and Mohtar 2013; Mays 2014). An unusual feature in highly developed arid countries like the Arabian Gulf countries is the use of lagoons as artificial lakes for storing and discharging surface water, treated wastewater (TWW), and/or untreated wastewater. Most of those water-scarce countries such as Qatar use lagoons as storage for TWW because the recycling of that water is extremely limited due to the perceived cultural limitation (Al-Mohannadi 2004; Ibrahim and Alshaoui 2015). This has resulted in excess TWW mixing with waters from other sources accumulating in lagoons are left to evaporate or infiltrate into the groundwater as an indirect groundwater recharge. Globally, the environmental issues of wastewaters, TWW, and lagoons affecting groundwater reservoir bacterial communities have been reported in many studies. Most of them reported microbial groundwater contamination by untreated wastewaters and sewage seeping into groundwater (Antunes et al. 2019; Bamigboye et al. 2020; Castro et al. 2015), with relatively few reported TWW with secondary and tertiary lagoons’ water infiltration (Ofori et al. 2021; Popovic et al. 2015; Zaouri et al. 2020). Bacterial groups belonging to the total coliforms, fecal coliforms, and *E. coli* using selective media where the focus of few studies exploring groundwater contamination from wastewaters (Sanders et al. 2013; Talat et al. 2019). This is because groundwater globally is mostly reserved for drinking purposes. Thus, legislative standards use total coliforms, fecal coliforms, and *E. coli* bacteria to control groundwater pollution (Herschy 2012; U.S. Epa 2017). However, previous research has highlighted the importance of monitoring other bacteria which are known as TWW pathogenic bacteria, which are used as indicators of contamination of groundwater near wastewater or TWW sources. This was observed to be significantly higher in urban settlements and where the groundwater is adjacent to septic tanks and wastewater treatment plants (Andrade et al. 2020; McKeon et al. 1995; Zaouri et al. 2020), indicating the importance of extending the study beyond simply using coliform bacteria and *E. coli* only as indicators of bacterial contamination. Identification of the environmental microorganisms in water is possible through several methods, including ribotyping using the 16S rRNA (Chen et al. 2021; Sorensen et al. 2015; Suzuki et al. 2018) and the “matrix-assisted laser desorption/ionization time-of-flight mass spectrometry” (MALDI-TOF MS) (Ashfaq et al. 2019; Sala-Comorera et al. 2016; Suzuki et al. 2018; Tang et al. 2021). MALDI-TOF MS is an instrumental technology for rapidly generating protein profiles from living cell samples and has been widely used for bacterial identification and in proteomics for disease-related biomarker development using proteomic profiling and biomarker identification (Cobo et al. 2016; Tang et al. 2021). It has been recently used for studies of bacterial contamination in the environment for its accurate and rapid results in terms of bacterial flora identification in wastewater and groundwater (Lasch et al. 2025; Tang et al. 2021). Studies have shown that the MALDI-TOF MS identification success rate is approximately 96%, suggesting that it can be used as a tool to rapidly and effectively classify bacteria in water by protein profiling (Suzuki et al. 2018). Studies on bacterial communities in lagoons and nearby groundwater are scarce since most research concentrates on individual locations or small systems, typically examining either surface water or groundwater in isolation (Fridrich et al. 2014; Huang et al. 2021). Interactions between lagoons and aquifers are intricate, shaped by hydrology, nutrient dynamics, and seasonal changes, necessitating extensive, long-term, and multilocation monitoring that has rarely been undertaken. Microbial communities in aquatic and groundwater systems within arid regions such as Qatar are vital for nutrient cycling, maintaining water quality, and ensuring proper urban water management in a harsh arid environment. In addition, Qatar represents an ideal setting for studying bacterial community dynamics where lagoon settings and wells of groundwater are close to each other, since the country serves as a model for challenges related to water scarcity, environmental vulnerability, groundwater availability, agricultural development, and other interlinked sustainability issues. The research hypothesizes that TWW lagoons act as microbial reservoirs influencing groundwater bacteriomes through hydrological transfer. Hence, the study proposes a generalized and applicable strategy for managing microbial population dynamics before deploying lagoon systems and utilizing TWW for irrigation, considering the importance of lagoons as a central solution to overcome the persistent water demands in arid and semiarid environments.

## Material and methods

### Sampling sites and collection

**Fig. 1:**
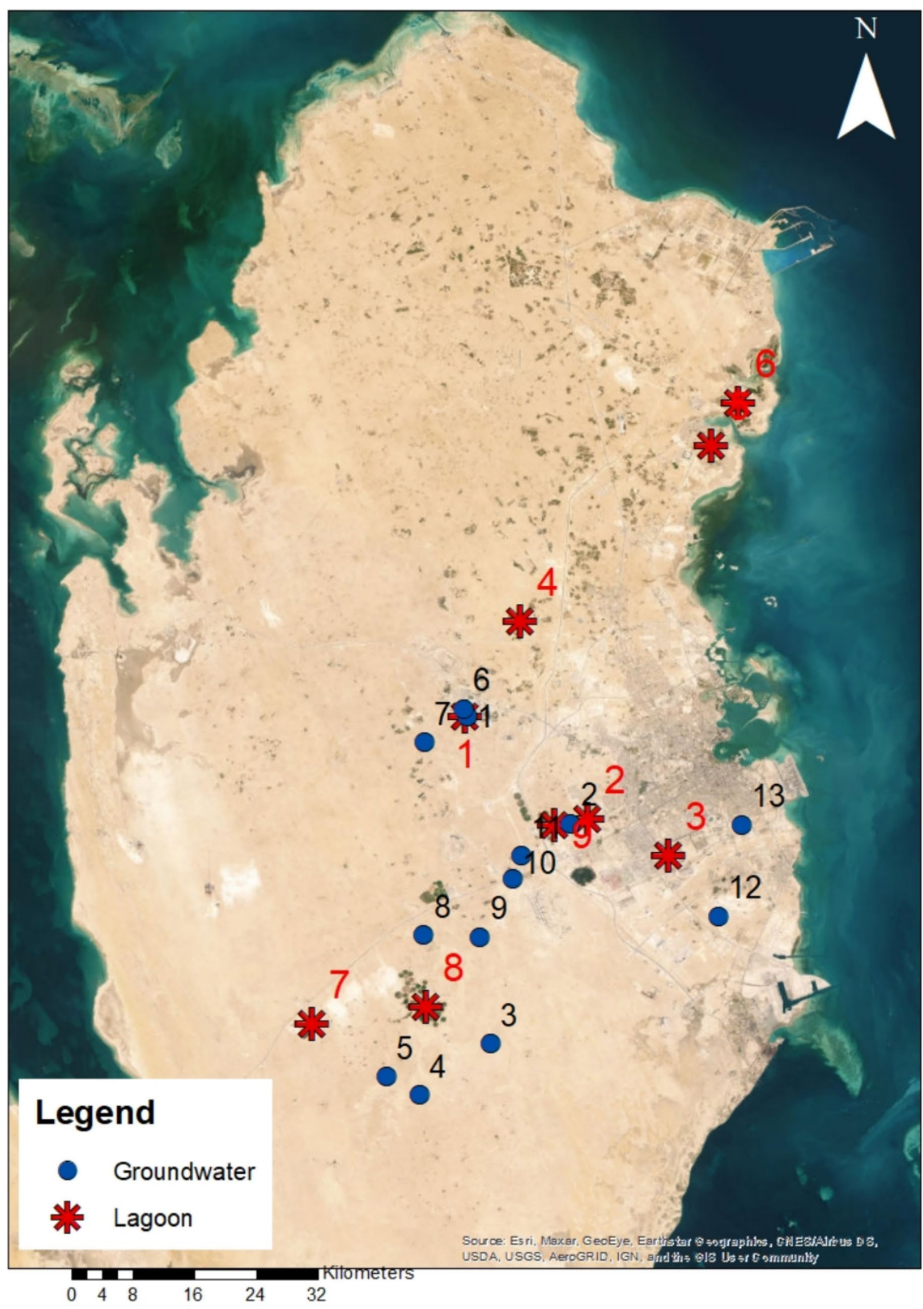
Map of Qatar showing the locations of sampled lagoons (red) and adjacent groundwater wells (blue)

Nine lagoons representing all artificial lagoons storing TWW in Qatar were identified from recent satellite images of Qatar using Google Maps, and 13 wells were selected based on their different proximity to the lagoons (0.5–10 km) to represent aquifers susceptible to lagoon water infiltration, as shown in Fig. 1. The sites were validated by a committee of five specialists from Qatar University and the University of Nottingham as interesting sites for the Qatari strategies. Water samples were collected in December–January 2022 in sterile tubes, transported at 4 °C, and analyzed on the same day.

### Microbial analysis

From each water sample, 100 µL was plated onto MacConkey and EMB agar to isolate Gram-negative faecal bacteria. Samples with high colony-forming units (CFU) counts were diluted, while those with low counts were concentrated by vacuum filtration onto 0.45-μm membranes before incubation. Individual colonies were purified through sequential streaking on selective agar to ensure culture purity. Purified isolates were preserved in LB liquid medium with 30% sterile glycerol at − 70 °C and later revived on LB agar for subsequent analyses.

### Identification of the isolated strains

Bacterial identification was performed using a Bruker MALDI Biotyper (Microflex LT). Pure colonies, from overnight incubation on solid LB medium, were transferred to MALDI target plates, overlaid with HCCA matrix, and analyzed within the range of 0–30,000 Da. Spectral profiles were compared to reference databases using Biotyper software, with log scores ≥ 2.0 considered reliable for species-level identification and those ranging between 1.7 and 1.99 accepted for genus-level identification. The MALDI-TOF MS generated both an identification dataset and a protein fingerprint dataset of each bacterial isolate for further analyses.

### Data processing and analysis of the similarity of the bacterial isolates

Heatmaps of CFUs related to abundances were generated in OriginPro to illustrate the bacterial distribution across sites. Protein mass spectra were preprocessed in R (MALDIquant) using smoothing, baseline correction, normalization, peak detection, and spectral alignment, resulting in an intensity-based profile. Strain-level similarities were visualized with a dendrogram. Principal component analysis (PCA) and generalized linear model (GLM) were applied to explore clustering patterns and test relationships between bacterial abundance, lagoon proximity, and groundwater contamination. Bacterial alpha diversity was assessed at each site using Shannon, Simpson, and Chao1 indices, calculated from CFU values (Chauhan et al. 2025). Mann–Whitney *U* tests were used to assess whether diversity and richness differed significantly between the lagoon and adjacent groundwater wells. All analyses were performed using MATLAB.

## Results and discussion

### Bacterial communities in both lagoon waters and wells’ groundwater

The colonies chosen from the plates with selective media in this study were selected mainly for their shape, size, color, cluster, and other visually unique characteristics. However, it is important to recognize that this strategy does not rule out the chance that some of the chosen colonies might be from the same bacterial genus, since observable variations do not necessarily align with genetic or taxonomic differences. The selective media of MacConkey and EMB were chosen to target Gram-negative bacteria, particularly *Enterobacteriaceae*, which are widely used as indicators of fecal contamination and water pollution. In the context of lagoons and their potential for infiltration into groundwater, these microorganisms are of particular interest due to their ability to survive, be transported, and persist in aquatic and porous environments. Their detection thus allows for the assessment of the risk of transfer of microbiological contaminants to groundwater. However, this approach remains targeted and may underestimate overall microbial diversity, which must be taken into account in further related studies. In this study, however, the targeted bacteria are those that can be fecal-infectious bacteria, the majority of which pose a health risk.

A total of 150 bacterial strains were isolated and purified and then subsequently identified using MALDI-TOF MS. The identification performances of bacteria were 91% for the lagoons and 86% for the groundwater. This performance is in alignment with other studies showing that MALDI-TOF MS performance in the identification of environmental bacteria is always around 90%. (Ae 2019; Imai et al. 2020; Tang et al. 2021). The MALDI-TOF scores of all the identified strains were above 1.7, with the majority exhibiting scores equal or above 2.0 for both lagoon and groundwater bacteria. A total of 79 bacterial strains from lagoons were identified, and 71 from the wells. The locations, strain names, and corresponding scores are presented in the supplementary material section. Across the lagoons and groundwater sites, 14 strains were unidentified. Those were labeled in the figures and tables as (no identification), which refers to isolates that generated MALDI-TOF MS spectra but did not achieve a reliable match in the reference database (log score < 1.7) and were therefore not assigned to a taxonomic identity.

The identified bacterial strains consisted of 33 different bacterial species that were identified both in the lagoons and groundwater. All the bacterial species were found to belong to the *Proteobacteria* phylum with only one species from *Bacteroidetes* phylum. Moreover, 32 out of the 33 bacterial species were *Gammaproteobacteria*. Within the *Gammaproteobacteria* phylum, four main genera were found, namely, *Pseudomonas*, *Acinetobacter*, *Aeromonas*, and *Enterobacter*. They represent 37%, 15%, 12%, and 10%, respectively, of the total strains. The other genus is represented by one or two bacterial species accounting for 26% of the identified bacterial strains.

### Comparative analysis of bacterial relative abundance in lagoons and groundwater

**Fig. 2:**
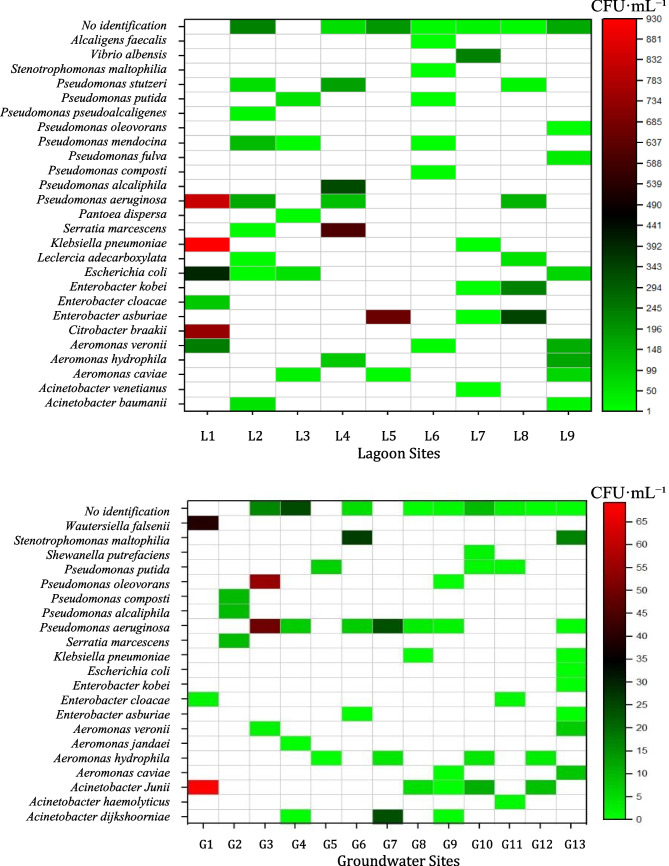
Heatmaps of bacterial strain abundance across nine lagoon sites (0–930 CFU mL⁻^1^) and 13 groundwater sites (0–70 CFU mL⁻^1^) in Qatar, illustrating higher abundance in lagoons and distinct community patterns between the two environments (L: lagoon; G: groundwater)

In order to compare and describe bacterial abundance in the two environmental settings, heatmaps were produced for both lagoons and groundwater (Fig. 2). It was clear that the bacterial species in lagoons, and the corresponding adjacent wells’ groundwater, were not homogeneously distributed. Indeed, the lagoons exhibited high bacterial diversity in terms of both abundance and species composition. The heatmap corresponding to lagoons was between 1 and 930 CFU mL^−1^, while that to the groundwater spanned between 1 and 70 CFU mL^−1^. Wastewaters usually exhibit higher numbers of Gram-negative bacterial strains than in groundwater as reported in previous studies (Malik and Aleem 2011; Xu et al. 2020). This trend was also observed in the lagoons and adjacent groundwater. Lagoon 2 and 9 showed the highest abundance in terms of total and unidentified numbers of bacterial species. Lagoon 9 is an evaporation lagoon intended for TWW from the primary wastewater treatment facility serving the areas around the capital, Doha. The effluent produced was reported to be 100% recycled in destinations including (i) Doha City which uses 42% of the TWW for irrigation of green areas, (ii) farms which use 55% of the water for irrigation in the desert areas, and (iii) aquifers which were replenished by injecting the remaining 3% (DWTP 2010). Lagoon 2 is situated in a government-protected area (MDPS 2019). This lagoon was reported to receive surface water from network leakage and rainfall events. Therefore, the water is dumped into the lagoon without any treatment. The least number of bacterial species was from Lagoon 5. In fact, this lagoon is situated on the low-lying ground adjacent to the coast, where seawater may mix with the lagoon water, thus diluting it, in addition to the increase of salinity which both may cause further lowering of the bacterial abundance (Aljabiry). The remaining lagoons (1, 3, 4, 6, 7, and 8) showed similar abundance patterns in Fig. 2 and comparable alpha diversity indices (Table 1). These lagoons are located near treatment plants containing surplus TWW. Even after treatment, this water may retain nutrients (e.g., organic matter, nitrogen, and phosphorus), and the lagoon environmental conditions (such as retention time, sedimentation, and water–sediment interactions) can contribute to the accumulation and diversification of bacterial strains. Thus, the observed patterns are likely linked to the origin of the TWW and further influenced by the lagoon-specific ecological characteristics.

**Table 1:** Alpha diversity indices (Shannon *H*′, Simpson *D*, and Chao1 richness estimator) of bacterial communities detected in different lagoon sites in Qatar, showing variation in species diversity and richness among the sites

**Site**	Shannon index (*H*′)	Simpson index (*D*)	Chao1
Lagoon 1	1.603	0.778	6
Lagoon 2	1.719	0.779	9
Lagoon 3	1.457	0.742	5
Lagoon 4	1.500	0.724	6
Lagoon 5	0.626	0.38	3
Lagoon 6	1.729	0.801	7
Lagoon 7	0.561	0.24	6
Lagoon 8	1.352	0.694	6
Lagoon 9	1.812	0.816	8
Well 1	0.662	0.464	3
Well 2	1.099	0.667	3
Well 3	1.068	0.62	4
Well 4	0.592	0.358	4
Well 5	0.177	0.082	2
Well 6	0.903	0.49	4
Well 7	0.911	0.568	3
Well 8	0.937	0.55	4
Well 9	1.583	0.761	6
Well 10	1.308	0.685	5.5
Well 11	1.352	0.731	5
Well 12	0.663	0.405	3
Well 13	1.282	0.655	8

The well with the highest relative bacterial abundance was well 13, which is in the industrial area of Doha, where the anthropological activities are concentrated. The other wells exhibited more or less the same relative abundance of *P. aeruginosa*, *A. hydrophila*, and *A. junii* as the most abundant bacterial species. By comparing the relative bacterial abundances of the lagoons with those of the groundwater, it can be concluded that strains of the *Acinetobacter* species found in the lagoons (*A. baumannii* and *A. venetianus*) are different from the *Acinetobacter* strains in groundwater (*A. dijkshoorniae*, *A. haemolyticus*, and *A. junii*). Moreover, four species of *Pseudomonas* were found in lagoons but not in groundwater (*P. fulva*, *P. mendocina*, *P. pseudoalcaligenes*, and* P. stutzeri*). On the other hand, the species *P. aeruginosa* was characterized by the highest abundance in both lagoons and groundwater samples. Analysis using the results of the heatmap revealed notable variations in the bacterial community composition and abundance across all lagoons and groundwater samples, reflecting the impact of anthropogenic activities and treatment processes among wastewater treatment plants on the resulting microbial profiles. This was demonstrated in previous literature, either due to varying chemical composition or temporal microhabitat variation affecting bacterial growth (Laque et al. 2010; Mohit et al. 2014).

Statistical comparison of alpha diversity indices in Table 1 shows that lagoons exhibited significantly higher bacterial diversity and richness than groundwater wells (Shannon, *p* = 0.038; Simpson, *p* = 0.045; and Chao1, *p* = 0.012). These results indicate that lagoon environments support more diverse and evenly distributed bacterial communities, while groundwater wells are comparatively less diverse and dominated by fewer species. Lagoons generally symbolize dynamic aquatic ecosystems with varied microhabitats and increased nutrient levels. This environmental diversity fosters numerous bacterial species by offering a wide range and varied resources, such as organic matter and nutrients, that enhance microbial growth and diversity. In comparison, groundwater environments tend to be more stable yet lack nutrients, restricting the diversity and abundance of growing bacteria, resulting in communities primarily composed of fewer specialized species suited for low-nutrient conditions. Moreover, lagoons frequently encounter variable oxygen levels because of mixing, photosynthesis, and the decomposition of organic material, which establishes environments for both aerobic and anaerobic bacteria. This variability encourages the coexistence of various bacterial groups that fulfil distinct metabolic functions. In contrast, groundwater wells usually exhibit more consistently low oxygen or anoxic conditions, which benefits bacteria that thrive in such restrictive environments, leading to decreased community diversity and evenness. In addition, in lagoons, bacterial richness and diversity foster complex interaction networks that stabilize community structure via species cooperation or competition. In groundwater, less complex networks with fewer species lead to simpler communities that are less stable but characterized by particular keystone taxa suited to the distinct subsurface environment. These potential sources of differentiation are supported by the bacterial taxa isolated from lagoons and groundwater, including *Pseudomonas* and *Acinetobacter*. These bacteria are recognized for their exceptional ability to adapt to various and frequently harsh microenvironmental conditions, allowing them to surpass other bacterial species and grow in multiple settings. *Pseudomonas* species can transition between motile planktonic forms and sessile communities that form biofilms. *Acinetobacter* species can endure harsh and fluctuating environments, including nutrient-limited conditions. As the sampling was performed in December–January, these results represent the lagoons and groundwater microbial dynamics in the winter season. Providing an opportunity for future studies examining the temporal variation affecting the microbial communities of the two matrices.

### Identification of coliform bacteria and distribution patterns in lagoons and groundwater

Coliforms are generally represented by 4 genera: *Escherichia*, *Klebsiella*, *Citrobacter*, and *Enterobacter*. While not all coliform bacteria are harmful, their presence in water serves as an indicator of possible contamination by pathogenic bacteria originating from human waste (Bai et al. 2022). In this study, the identified bacterial strains by MALDI-TOF MS which were previously reported as coliform bacteria, are: *C. braakii*, *E. asburiae*,* E. cloacae*, *E. kobei*, *E. coli*, *K. pneumonia*, *L. adecarboxylata*, *S. marcescens*, and *P. dispersa* (Adapa et al. 2019; Alvarez-Guzmán et al. 2020; Bamigboye et al. 2020; Fedeila et al. 2018; Gutierrez and Montalla 2020). The cell counts (CFU mL⁻^1^) of coliform bacteria, summarized in Table 2, show marked fecal contamination of the lagoons, with coliform counts ranging from 27 to 2410 CFU/mL, or approximately 2700 to 241,000 CFU/100 mL. These values far exceed the drinking water standards recommended by the WHO and EPA, which require the absence of *E. coli *in 100 mL of drinking water. They also exceed the reference values used for the unrestricted reuse of wastewater for irrigation, generally set at 1000 fecal coliforms/100 mL. The wells show lower levels of contamination but still do not meet microbiological drinking water standards, suggesting a possible influence of surface water or environmental inputs on the aquifer quality. The wells showed significantly lower coliform levels than the lagoons, but several samples still failed to meet microbiological drinking water standards. Indeed, WHO and EPA guidelines require the absence of detectable coliforms/*E*. *coli* in 100 mL of drinking water. Thus, although the groundwater showed lower contamination, it cannot be considered safe for consumption without further treatment.

**Table 2:** Total coliform bacterial cell counts in lagoons and groundwater, with the maximum allowable standards for Qatar and WHO quality standards for TWW

Location	Total coliform bacteria CFU mL^−1^
Lagoon 1	2410
Lagoon 2	250
Lagoon 3	87
Lagoon 4	160
Lagoon 5	867
Lagoon 6	27
Lagoon 7	33
Lagoon 8	630
Lagoon 9	633
Well 1	0.1
Well 2	10
Well 3	18
Well 4	25
Well 5	0.3
Well 6	5
Well 7	4
Well 8	0.7
Well 9	2
Well 10	14
Well 11	3
Well 12	3
Well 13	17
Qatar crop irrigation guidelines^1^	2.2 × 10^–2^
Qatar landscaping irrigation guidelines^2^	23 × 10^–2^
WHO TWW discharge standards^3^	10

^1^Qatar quality standards for TWW used for irrigation, maximum allowable for food crops (Ras Laffan Industrial City 2020)

^2^Qatar quality standards for TWW used for irrigation, the maximum allowable for landscaping (Ras Laffan Industrial City 2020)

^3^WHO maximum allowable standards for TWW used for irrigation coliform bacteria spatial abundance in relation to other water quality variables (WHO 2017)

### Bacterial community relationship across lagoons and the adjacent groundwater

**Fig. 3:**
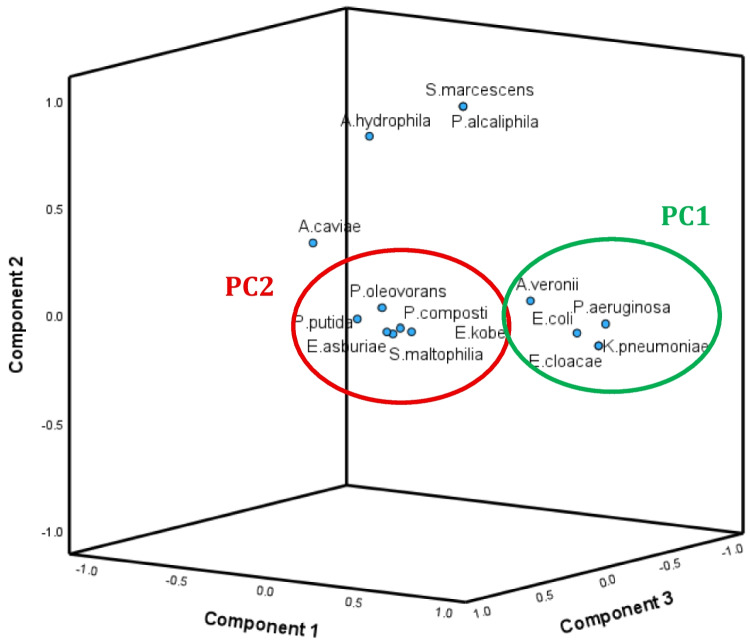
A 3D PCA plot illustrating the distribution of MALDI-TOF MS-identified bacterial strains based on their abundance (CFU mL⁻^1^), revealing distinct clustering patterns along PC1 and PC2

PCA was the major statistical tool which was previously used to explore the relationship between several bacteria of interest in contaminated waters and the chemical characteristics (Korbel et al. 2022; Mouser et al. 2010) or the occurrence of bacterial indicators in different locations influenced by their microhabitat (Akita et al. 2021; Gonzalez et al. 2010). In this research, PCA was conducted between the isolated and identified bacterial strains and their abundance in terms of cell counts (CFU mL^−1^). It was performed using varimax rotation and the data were standardized (*z*-score) prior to PCA to account for differences in scale. The PCA produced 3 PCs, i.e., PC 1 (32%), PC 2 (17%), and PC 3 (12%) that cumulatively explain 61% of the variations (KMO = 0.688, and Bartlett’s < 0.001). Results from PCA showed that the bacterial species are divided into two groups (Fig. 3) with PC1 illustrating the cluster of *A. veronii*, *E. cloacae*, *E. coli*, *K. pneumoniae*, and *P. aeruginosa* representing facultative anaerobic bacteria in TWW, suggesting their similar behavior and occurrence across both lagoon and groundwater environments. PC2 allowed clustering of *A. hydrophila*, *E. kobei*, *E. asburiae*, *S. maltophila*, and *P. oleovorans*, which are mostly facultative anaerobes but not necessarily TWW-related.

To further assess the influence of lagoon location and proximity on coliform bacteria abundance in groundwater, a GLM analysis was applied using log-transformed coliform bacteria counts (log₁₀ CFU·mL⁻^1^) in groundwater wells as the dependent variable and distance to the nearest lagoon as the predictor. The analysis showed that coliform bacteria abundance showed a marginal, nonsignificant decrease with distance (*p* = 0.061). However, when using the CFU for TWW-bacteria indicators, i.e., *A. veronii*, *E. cloacae*, *E. coli,* *K. pneumoniae*, and *P. aeruginosa,* which have been reported in previous literature as bacterial biomarkers for WW pollution (Chen et al. 2019; McKeon et al. 1995; Padhi et al. 2013), GLM analysis revealed a significant negative effect of the distance between groundwater (*B* = −0.157, *p* = 0.008), corresponding to an approximate 30% decrease in bacterial abundance per kilometer. This pattern indicates that wells located closer to lagoons contain higher levels of these bacteria, supporting the role of lagoons as potential sources of microbial infiltration into groundwater systems.

### Investigation of bacterial strain similarities based on protein profiles using PCA

**Fig. 4:**
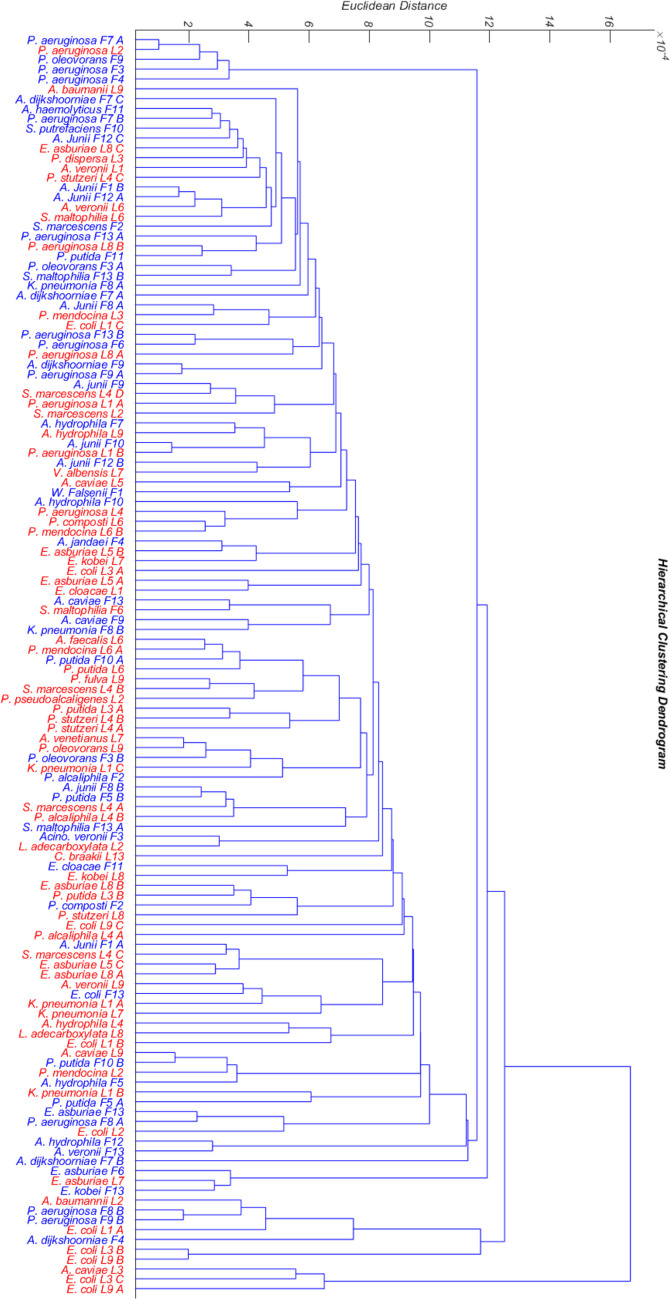
Dendrogram for the preprocessed MALDI-TOF MS protein profiles of the 150 isolated strains from lagoons and groundwater (lagoon in red and groundwater in blue)

Few studies have successfully linked environmental bacteria to the potential contamination source (Ae, 2019; Akita et al. 2021; Lyons et al. 2021; Santos et al. 2018). Employing MALDI-TOF MS protein profiles as bacterial fingerprints to associate biomarkers with contamination sources is innovative, as it enables swift, precise, and high-resolution identification of bacteria through distinct protein patterns. This method surpasses conventional techniques by linking particular bacterial protein markers to environmental pollution, improving microbial source tracking with efficiency and accuracy (Lasch et al. 2025; Santos et al. 2016; Suzuki et al. 2018). Thus, the extracted and preprocessed protein profiles for the 150 isolated strains from lagoons and groundwater were subjected to dendrogram analysis to cluster the strains according to their protein expressions. The dendrogram produced in Fig. 4 shows that there is no distinctive pattern of clustering between the isolated bacteria. However, most of the isolated strains of *E. coli* and *P. aeruginosa* from both lagoons and groundwater were grouped in the same clade, with *P. aeruginosa* forming a separate clade distinct from all the other strains. The *E. coli* strains isolated from lagoons 3 and 9 are clustered together. They are of interest as those lagoons are sourced from two different plants treating wastewater from the capital, Doha, with similar treatment processes. This may explain the similarity between their isolated *E. coli* strains. The clustering of *P. aeruginosa* suggests that this strain likely originates from surface environments within different lagoons and subsequently infiltrates into the groundwater system. This has been demonstrated in several studies highlighting the persistence, accumulation, and infiltration of *P. aeruginosa* from various contamination sources into groundwater aquifers (Nasreen et al. 2015; Toze 2003; Wang et al. 2023), emphasizing the importance of including this strain in the microbial standards for assessing water quality in Qatar.

**Fig. 5:**
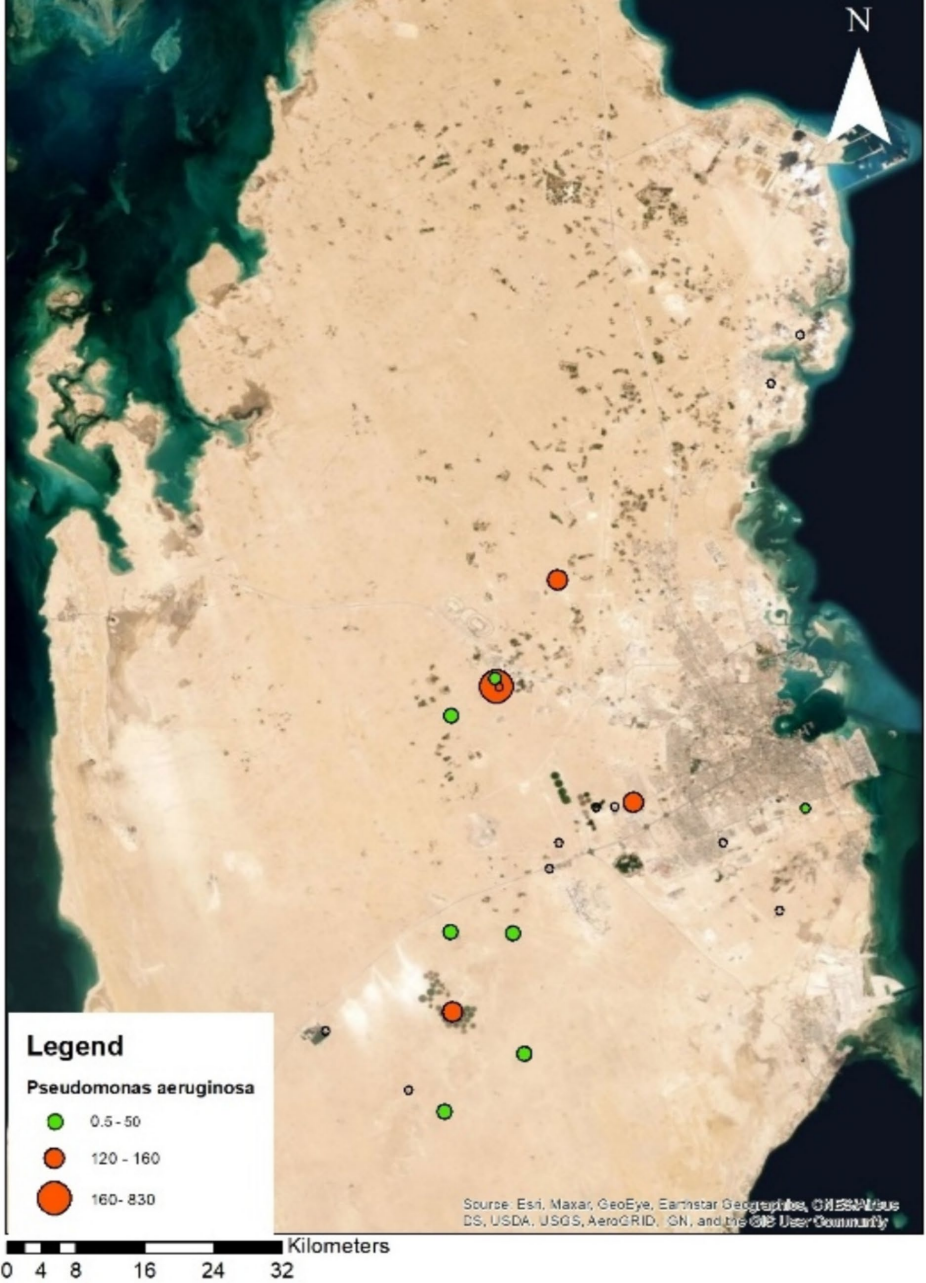
Spatial distribution of *Pseudomonas aeruginosa* in lagoons (red) and adjacent groundwater (green) across Qatar (CFU mL⁻^1^)

To further assess the abundance of *P. aeruginosa*, a distribution map shown in Fig. 5 illustrates the higher abundance of the strain in the lagoons along with its occurrence in the adjacent wells. *P. aeruginosa* is a well-known human pathogen causing hospital-acquired infections, especially respiratory and urinary tract infections in immunocompromised patients (Bentzmann and Plésiat 2011; Slekovec et al. 2012). In Qatar, this bacterium was considered to have the most cases of infection in cystic fibrosis patients (Abdul Wahab et al. 2005; Wahab et al. 2004). Studies have shown that this pathogen is found in WW and develops high resistance to antibiotics, classifying it as a “super bug” that finds its way into tap water. Moreover, a few studies recommend that this bacterium should be monitored and regulated similarly to *E. coli* and fecal bacteria in drinking water (Al-Ansari et al. 2021; Petit et al. 2013; Slekovec et al. 2012).

Similar results had also been discussed in previous literature, exploring the use of noncoliform bacteria as biomarkers for wastewater impact on groundwater aquifers (Zlender and Rupnik 2023). WHO guidelines for drinking water have highlighted the importance of monitoring noncoliform bacteria alongside fecal bacteria and *E. coli* (Herschy 2012). Previous literature reports that used protein profiles have acknowledged the capacity of MALDI-TOF MS to categorize strain isolates by their potential source, making it a tool for fingerprinting environmental isolates (Siegrist et al. 2007; Giebel et al. 2008; Santos et al. 2016; Alsayegh et al. 2021). This leads to the conclusion that the bacterial strains found in groundwater that correlate to the strains found in lagoons, following protein profile expressions, probably originate from TWW. Traditional coliform indicators primarily reflect recent fecal contamination but may not capture persistent, opportunistic pathogens associated with TWW systems. In this study, the consistent detection of *Pseudomonas aeruginosa* across both lagoons and adjacent groundwater highlights its potential as a robust biomarker of wastewater infiltration. These findings highlight the importance of expanding microbial water quality guidelines to include TWW-associated bacteria, with particular emphasis on *P. aeruginosa,* within groundwater monitoring frameworks in Qatar. Incorporating such indicators can support early detection of contamination and improve management strategies aimed at mitigating the hydrological transfer of TWW into groundwater aquifers. Beyond conventional microbiological contamination, a major emerging risk is the transfer of antibiotic resistance via groundwater influenced by lagoons. Lagoons can act as reservoirs of resistant bacteria and resistance genes, which can then be mobilized by infiltration into the soil and groundwater. Thus, even when conventional water quality indicators decrease with distance or depth, the risk of resistance dissemination can persist in the environment. This problem is particularly concerning in wastewater reuse systems, where microbial load and selection pressure can promote the spread of resistance determinants. Hence, future work on the hydrological transfer of microbial pollutants should include antibiotic-resistant bacteria for better water management.

## Conclusion

This study represents the application of MALDI-TOF MS to investigate bacterial communities in lagoons and adjacent groundwater in Qatar. The approach successfully identified Gram-negative bacteria and revealed that lagoons hosted more diverse and evenly distributed bacterial communities compared to wells, which were less diverse and dominated by fewer species. The results showed strong associations between coliform bacteria and TWW-associated strains such as *Escherichia coli*, *Enterobacter cloacae*, *Klebsiella pneumoniae*, *Aeromonas veronii*, and particularly *Pseudomonas aeruginosa*. The clustering of *P. aeruginosa* across lagoons and wells highlights its potential role as a biomarker of lagoon infiltration into groundwater. These findings provide important evidence of the importance of frequent monitoring and MALDI-TOF MS application in urban water management of TWW and its impact on groundwater quality in arid regions and support the need to include *P. aeruginosa* alongside coliforms in future monitoring and water reuse regulations in Qatar.

## Supplementary Information

Below is the link to the electronic supplementary material.

ESM 1 Supplementary Material 1 (DOCX 19.2 KB)

## Data Availability

Data will be made available on request.
