# Supplementary material for: Bacterial community profiling in artificial lagoons and groundwater of Qatar using a MALDI-TOF MS approach
Source: Environ Sci Pollut Res Int. 2026 May 29;33(18):8957–70. doi: 10.1007/s11356-026-37851-4 (PMC13253597; doi:10.1007/s11356-026-37851-4)
Supplement: Supplementary file 1 — Supplementary Material 1 (DOCX 19.2 KB) [file 11356_2026_37851_MOESM1_ESM.docx]

**Supplementary Materials**

**Journal of Environmental Science and Pollution Research**

**Bacterial Community Profiling in Artificial Lagoons and Groundwater of Qatar using A MALDI-TOF MS Approach**

*Senior and Corresponding Author: Hayat Aljabiry Email: [haljabiry@qu.edu.qa](mailto:haljabiry@qu.edu.qa)

Qatar University. PO. Box 2713, Doha, Qatar.

**Table 1.** Identified bacterial strains from lagoons, along with their corresponding identification scores, given inside brackets.

| **Lagoon 1** | **Lagoon 2** | **Lagoon 3** | **Lagoon 4** | **Lagoon 5** | **Lagoon 6** | **Lagoon 7** | **Lagoon 8** | **Lagoon 9** |
| --- | --- | --- | --- | --- | --- | --- | --- | --- |
| Aeromonas veronii (2.21)  Enterobacter cloacae (2.3)  Escherichia coli (2.3)  Klebsiella pneumonia (2.11)  Escherichia coli (2.02)  Pseudomonas aeruginosa (2.59)  Klebsiella pneumonia (2.36)  Escherichia coli (2.25)  Pseudomonas aeruginosa (2.34)  Klebsiella pneumonia (2.33)  Citrobacter braakii (2.23) | *Pseudomonas aeruginosa* (2.34)  *Pseudomonas mendocina* (1.97)  *Escherichia coli* (2.33)  no identification (0)  *Acinetobacter baumanii* (2.46)  *Pseudomonas stutzeri* (2.26)  *Pseudomonas pseudoalcaligenes* (2.23)  *Leclercia adecarboxylata* (2.23)  *Serratia marcescens* (2.33) | Aeromonas caviae (2.32) Pseudomonas putida (2.3) Pseudomonas mendocina (216) Pantoea dispersa (2.44) Escherichia coli (2.31) Escherichia coli (2.21) Escherichia coli (2.23) Pseudomonas putida (2.3) | Pseudomonas aeruginosa (2.53) Aeromonas hydrophila (2.13) Serratia marcescens (2.21) Pseudomonas stutzeri (2.3) Pseudomonas stutzeri (2.26) Pseudomonas stutzeri (2.08) Serratia marcescens (2.27) Pseudomonas alcaliphila (2.17) Serratia marcescens (2.34) Pseudomonas alcaliphila (2.29) Pseudomonas (1.48) Serratia marcescens (2.23) | Aeromonas caviae (2.39) Enterobacter asburiae (2.23) Enterobacter asburiae (2.11) no identification (0) Enterobacter asburiae (2.27) | Stenotrophomonas maltophilia (2.04) Pseudomonas mendocina (2.19) Pseudomonas mendocina (3.19) Aeromonas veronii (2.3) Pseudomonas composti (2.16) Alcaligenes faecalis (2.16) Pseudomonas putida (2.19) | Enterobacter (167) Pseudomonas (169) Vibrio albensis (1.95) Enterobacter kobei (2.25) Klebsiella pneumonia (2.3) Acinetobacter venetianus (2.32) Enterobacter asburiae (2.32) | no identification (0) Pseudomonas aeruginosa (2.5) Pseudomonas aeruginosa (2.47) Pseudomonas stutzeri (2.11) Enterobacter asburiae (2.17) Enterobacter kobei (2.07) Leclercia adecarboxylata (2.4) Enterobacter asburiae (2.32) Enterobacter asburiae (2.28) | Escherichia coli (2.21) no identification (0) Aeromonas caviae (2.28) Aeromonas hydrophila (2.23) Aeromonas veronii (2.24) no identification (0) Escherichia coli (2.32) Escherichia coli (2.24) Pseudomonas fulva (2.02) Acinetobacter baumanii (2.38) Pseudomonas oleovorans (1.73) |

**Table 2.** Identified bacterial strains from groundwater, along with their corresponding identification scores, given inside brackets.

| **Well 1** | **Well 2** | **Well 3** | **Well 4** | **Well 5** | **Well 6** | **Well 7** | **Well 8** | **Well 9** | **Well 10** | **Well 11** | **Well 12** | **Well 13** |
| --- | --- | --- | --- | --- | --- | --- | --- | --- | --- | --- | --- | --- |
| Acinetobacter junii (2.32) Wautersiella falsenii (2.23) Acinetobacter junii (2.49) | Pseudomonas alcaliphila (1.92) Pseudomonas composti (1.87) Serratia marcescens (2.3) | Acinetobacter veronii (2.02) Pseudomonas oleovorans (1.81) Pseudomonas oleovorans (1.78) Pseudomonas aeruginosa (2.3) | Aeromonas jandaei (2.11) Pseudomonas (1.58) Pseudomonas aeruginosa (2.37) Acinetobacter dijkshoorniae (1.86) | Aeromonas hydrophila (2.13) Pseudomonas putida (2.4) Pseudomonas putida (2.33) | Enterobacter asburiae (1.86) no identification (0) Stenotrophomonas maltophilia (2.01) Pseudomonas aeruginosa (2.54) | Aeromonas hydrophila (2.27) Pseudomonas aeruginosa (2.41) Acinetobacter dijkshoorniae (1.98) Acinetobacter dijkshoorniae (2.02) Acinetobacter dijkshoorniae (1.93) Pseudomonas aeruginosa (2.42) | Pseudomonas aeruginosa (2.36) Klebsiella pneumonia (2.1) Acinetobacter junii (2.3) no identification (0) Klebsiella pneumonia (2.1) Acinetobacter junii (2.38) Acinetobacter junii (2.46) Pseudomonas aeruginosa (2.38) | Aeromonas caviae (2.34) Pseudomonas oleovorans (1.96) no identification (0) Acinetobacter junii (2.5) Pseudomonas aeruginosa (2.44) Pseudomonas aeruginosa (2.35) Acinetobacter dijkshoorniae (2.2) | Pseudomonas putida (2.34) Aeromonas hydrophila (2.22) no identification (0) Pseudomonas putida (2.13) Acinetobacter junii (2.42) Shewanella putrefaciens (1.82) | no identification (0) no identification (0) no identification (0) Acinetobacter haemolyticus (2.39) Pseudomonas putida (1.81) Enterobacter cloacae (2.13) | Acinetobacter junii (2.32) Aeromonas hydrophila (2.2) Enterobacter (1.66) Acinetobacter junii (2.34) Acinetobacter junii (2.28) | no identification (0) Aeromonas veronii (2.22) Escherichia coli (2.27) no identification (0) Aeromonas caviae (2.18) Pseudomonas aeruginosa (2.59) Enterobacter kobei (1.96) Stenotrophomonas maltophilia (2.02) Pseudomonas aeruginosa (2.3) Enterobacter asburiae (2.17) Stenotrophomonas maltophilia (2.03) |
